# Effect of Matrine on HPAC cell migration by down-regulating the expression of MT1-MMP via Wnt signaling

**DOI:** 10.1186/s12935-015-0210-4

**Published:** 2015-06-11

**Authors:** Yongchao Ma, Fazhang Zou, Junping Xiong, Wei Wan, Li Yin, Xianjia Li, Zhanyu Bei, Lei Yuan, Song Meng, Jianguo Wang, Guohua Song

**Affiliations:** Luohe Medical College, Daxue Road, #148, Luohe City, Henan Province 462000 People’s Republic of China; Key lab, of BioMedicine of Luohe City, Luohe, China; Chengde Nursing Vocational College, Chengde, China

**Keywords:** Matrine, Cell migration, MT1-MMP, Wnt, Human pancreatic cancer

## Abstract

**Aim:**

This study sought to explore the exact mechanism of Matrine inhibited migration and invasion of human pancreatic cancer cells.

**Methods:**

HPAC or Capan-1 cells were cultured in completed RPMI-1640 medium, contained with 50 μg/ml Matrine or 0.05 μg/ml docetaxel, respectively. Cell viability was evaluated by spectrophotometric analysis using MTT assay. Wound healing assay and transwell approach were used to detect the effects of Matrine on HPAC cell migration and invasion. Western Blot and RT-PCR were performed to detect the expressions of MT1-MMP, Wnt and β-Catenin. CHIP assay was used to detect whether the MT1-MMP transcription activity correlated with Wnt signaling pathway.

**Results:**

MTT results indicated that cell proliferration was inhibited by Matrine at a range of concentrations, especially at high dose. We further found that Matrine treatment significantly induced cell migration and invasion decreased. Interestingly, the expression of MT1-MMP decreased evidently upon Matrine treatment, paralleled with the expressions of Wnt and β-Catenin detected by Western Blot and RT-PCR assay. Further analysis of MT1-MMP transcription activity revealed that Matrine reduced the expression of MT1-MMP mediated by Wnt signaling pathway.

**Conclusion:**

Matrine play a vital role in inhibiting HPAC cellular migration and invasion through down-regulating the expression of MT1-MMP via Wnt signaling pathway.

**Electronic supplementary material:**

The online version of this article (doi:10.1186/s12935-015-0210-4) contains supplementary material, which is available to authorized users.

## Introduction

Matrine has been used for cancer treatment in China for a long time, which was extracted from Chinese Medicine plant. It was demonstrated that matrine was efficiency and safety using in clinic. Previous researches reported that Matrine has a role in inhibiting the growth of tumor cells directly [1]. In clinical work it has been used for treating the uterine cervical cancer and leukemia [2, 3]. Recently, some researchers suggest that matrine could inhibit cancer cells migration, invasion and metastasis [4–6]. But the exact molecular mechanism of matrine in inhibiting cell adhesion and migration need further study. It was important to make an intensive investigation of the pharmacologic and clinic applications of matrine and maybe it could help researcher figure out the mechanism of tumorigenesis in human.

Wnt signaling pathway play a vital role in normal developments but also in tumorigenesis [7]. Inappropriate activation of Wnt signaling pathway results in the onset of several types of canceres [8]. Based on the differences of interactions with Wnt receptors or co-receptors, Wnt signaling pathway has been divided into three signaling pathways, namely the canonical Wnt/β-catenin, the non-canonical (or heretical) Wnt/ Ca^2+^ and planar cell polarity (PCP) pathways [9]. Previous researches suggested that the Wnt/PCP pathway interaction with some key proteines related with cell polarization, motility, cancer cell migration and invasion [10, 11]. β-catenin involved in the canonical Wnt pathway as a key mediator, which can enter cell nucleus and associate with transcription factors Lef and Tcf, leading to the transcription of Wnt target genes [12, 13]. The stabilization of β-catenin was regulated by phosphorylated modification by GSK3β, followed by degradation via the proteasome. Abnormal activated Wnt/β-catenin pathway has been detected in a number of human tumors including lung, breast, cervical and liver, that is due to β-catenin lack of degradation and ultimately nuclear accumulation. In hepatocellular carcinoma, β-catenin accumulation has been linked to poorly differentiated, high proliferative activity and poor prognosis [14, 15]. The level of β-catenin is regulated by numerous proteins, which, if not regulated or expressed appropriately would account for increased β-catenin expression in cancer. By forming a complex with Tcf-4, β-catenin activates the transcription of target genes included MT1-MMP, which correlates with cancer cell migration and invasion [16].

Abnormal expression of MT1-MMP has been found in many types of canceres. Such an induction expression of MT1-MMP could be regulated by the Wnt/β-catenin signaling pathway. This is based on the observation that depletion of β-catenin in colorectal carcinoma SW480 cells resulting down-regulation expression of MT1-MMP [17]. In the present study, our group has found that in some concentration ranges, matrine has the effect of inhibiting the migration of pancreatic carcinoma HPAC or Capanc-1 cells.

## Materials and methods

### Cell culture and experimental reagents

Human pancreatic cancer cells, such as HPAC, Capan-1 (from American Type Culture Collection, Rockville, MD) were cultured in RPMI-1640 (Invitrogen) supplemented with 10 % fetal bovine serum (FBS) and 100 units/mL penicillin and 100 μg/mL streptomycin, in a 5 % CO_2_ humidified atmosphere at 37 °C. Matrine (50 μg/ml, Qilu Pharmaceutical Co., Ltd.) and docetaxel (0.05 μg/ml, Sigma, USA) were added into the medium when necessary as indicated in figure legends. Primary antibodies of Wnt, β-catenin, MT1-MMP, and β-actin were purchased from Santa Cruz Biotechnology (Santa Cruz, USA). Other reagents used in this study, such as Anti-mouse-IgG-HRP and Anti-Rabbit-IgG-HRP, were purchased from California Bioscience (California Bioscience, USA), Transwell Invasion Chambers were found from Promega, USA.

### MTT assay

MTT [3-(4,5-dimethylthiazolyl-2-yl)-2,5-diphenyltetrazolium bromide] (Sigma- Aldrich; St. Louis, MO) assay was used for evaluation the viability of HPAC or Capan-1 cells upon Matrine treatment. Total 1 × 10^4^ cells/well were grown overnight in 96-well plate. The second day, various concentrations of Matrine were added and incubation for 48 h. MTT solution was added to each well at a final concentration of 500 μg/ml for 4 h. Formazan crystals formed by living cells were then dissolved in DMSO and measured at 570 nm by Multi-Detection microplate reader (Bio-Rad, USA).

### Monolayer cell migration assay

A monolayer wound-healing model was performed as cell migration assay. HPAC or Capan-1 cells were seeded in 6-well-plate for 24 h in RPMI-1640 medium. A confluent monolayer of HPAC cells were then scraped with a sterile 200 μl pipette tip and washed with PBS. After incubation with completed RPMI-1640 alone or contained with matrine (50 μg/ml) or with docetaxel (0.05 μg/ml) for 48 h, cell migration images were captured using an inverted phase contrast microscope at 100× magnification.

### Transwell invasion assay

Matrigel Invasion Chambers were hydrated for 4 h before starting the invasion assay. Log-phase cells (4 × 10^4^) were plated in 200 μl RPMI-1640 containing 2 % FBS in the upper chamber of the transwell. The lower chamber was filled with 500 μl completed RPMI-1640 containing 10 % FBS. The upper chamber cells were treated with matrine and docetaxel as previous description and allowed to migrate for 10 h at 37 °C and 5 % CO_2_ circumstance followed by carrying out the invasion assay. The cells were fixed for 15 min at room temperature by replacing the culture medium in the bottom with 4 % formaldehyde dissolved in PBS. Then, the cells on the upper chamber were moved with a cotton swab. After washing the chambers 5 times by dipping the chambers in a large beaker filled with dH_2_O, the cells that remained on the bottom of the lower chamber were stained with 0.1 % crystal violet. The migrated clones were photographed under an optical microscope. The cell numbers were counted at 12 different areas.

### RT–PCR analysis

Total RNA was extracted from HPAC cells using the TRIZOL reagent (Invitrogen Life Technologies). Reverse transcription (RT) was performed with 1 μg of total RNA and 10 μM of specific primers. cDNAs were amplified by polymerase chain reaction (PCR) for testing MT1-MMP (sense 5′-AGCCCCGAAGCCTGGCTACA-3′; antisense 5′-GCCGCCCTCACCATCGAAGG-3′,492-bp product) and Glyceral dehyde-3-phosphate dehydrogenase (GAPDH) (sense 5′-ACCACAGTCCAT GCCATCAC-3′; antisense 5′-TCCACCACCCTGTTGCTGTA-3′, 556-bp product), which was used as loading controls.

### Enzyme-linked immunosorbent assay

HPAC cells were treated as described above. Concentrations of MMP-9 and MMP-2 in the cell culture supernatants were quantified using MMP-9 and MMP-2 ELISA kits (R&D Systems, USA). Each sample was analysed in triplicate and manipulated according to the kit’s protocols.

### Western blot analysis

HPAC cells were lysed in RIPA buffer [50 mM Tris (pH 7.4), 150 mM NaCl, 1 % Triton X-100, 0.1 % SDS, 1 % sodium deoxycholate, 5 mM EDTA, 100 mM NaF, and 1 mM Na3VO4] containing a protease inhibitor cocktail for 30 min on ice, followed by centrifuged for 30 min at 16000 g. Protein concentrations were determined by the BCA method (Pierce, USA). Equal total proteins were electrophoresis by 12 % SDS-PAGE gel and transferred to PVDF membranes using a wet transblot system (Bio-Rad, Hercules, CA). The membranes were blocked for 1 h at room temperature with 5 % nonfat dry milk and incubated overnight at 4 °C with antibodies against Wnt, β-catenin, Axin, GSK-3β, MT1-MMP and β-actin (1:1000). After washing three times, membrane was incubated for 1 h with HRP-conjugated goat anti-rabbit secondary antibody diluted 1:5,000 in PBST. After further washing and processed using Super Signal West Pico chemiluminescent substrate (Pierce, USA), the membrane was exposed to Fujifilm LAS3000 Imager (Fuji, Japan). The band densities were normalized relative to the relevant β-actin with Image J Analyst software (NIH).

### Chromatine immuno-precipitation (CHIP) assay

CHIP assay was performed using the CHIP Kit (Sigma, USA) with slight modifications. HPAC cells (2 × 10^7^) were cross-linked with 1 % formaldehyde for 10 min at room temperature, followed by the addition of 1 ml of 125 mM glycine to inactivate the formaldehyde. Cells were washed twice with ice-cold PBS and then scraped and centrifuged at 1000 g at 4 °C for 5 min. Pelleted cells were lysed with 1 ml modified-RIPA lysis buffer (0.1 % SDS, 10 mM EDTA, 1 % Triton Χ-100 and 50 mM Tris–HCl pH 8.0) containing with protease inhibitor cocktail and incubated on ice for 10 min. After sonication to produce genomic DNA to lengths of 0.2 to 0.5 kb (optimized at 10 ~ 15-s pulses), samples were centrifuged at 13,000 g for 10 min to remove insoluble cell debris. Lysates were diluted in ChIP dilution buffer (0.01 % SDS, 0.1 % Triton X-100, 2 mM EDTA, 20 mM Tris–HCl pH 8.0 and 500 mM NaCl). Chromatin solution was precleared with 20 μl of 3 % BSA/protein A agarose for 2 h at 4 °C with rotation. Anti–β-catenin polyclonal (Santa Cruz Biotechnologies) antibody was added to the precleared supernatant and incubated overnight at 4 °C with rotation. Negative controls included a sample incubated without antibody and one incubated with rabbit IgG (Santa Cruz Biotechnologies) to determine whether interactions were due to nonspecific IgG interactions. Bead complexes were washed first with low-salt immune complex wash buffer, followed by high-salt immune complex wash buffer and a final LiCl immune complex wash buffer for 5 min each on a rotating platform followed by brief centrifugation. After the final wash, DNA was extracted by incubating the beads for 15 min with 200 μl elution buffer (1 % SDS and 50 mM NaHCO_3_). Samples were then uncrosslinked in a 65 °C water bath overnight, and DNA was purified using Qiagen Nucleotide Removal Kit (Qiagen, Valencia, CA). Polymerase chain reaction (PCR) primers used to amplify the MT1-MMP promoter region were as follows: GTCTCCCGCCCCAAGACCCT (forward) and GGAACACCACATCGGGGGCG (reverse).

### Statistical analysis

All experiments were performed three times and the results were expressed as mean ± SD. Statistical analysis was performed by SPSS11.0. *T* test was used in order to compare the average values between two populations of data. A *P* value of less than 0.05 was considered to indicate statistical significance.

## Results

### Effects of Matrine on migration and invasion of HPAC and Capan-1 cells

**Fig. 1:**
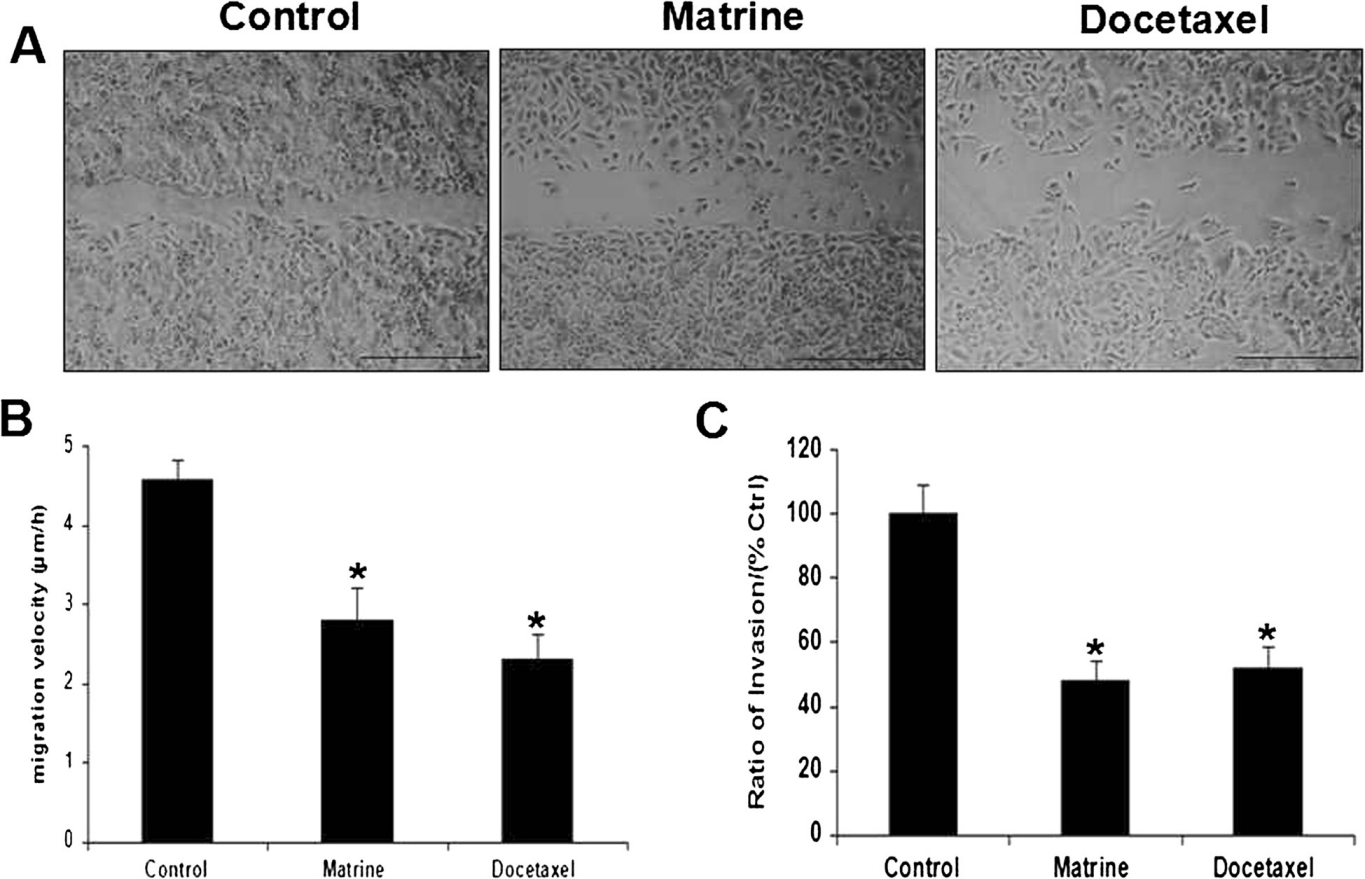
The migration of HPAC cells was inhibited by Matrine. Log-phase cells were treated with normal complete RPMI-1640 alone or contained with 50 μg/ ml Matrine or 0.05 μg/ ml Docetaxel (**a**). Data were expressed as mean ± S.E.M from three separated experiments (**b**). Cell invasion ability was detected by transwell assay (**c**). Statistical analyses was performed using the *t*-test. * (*P* < 0.05) indicates a significant difference compared with the control group

The effects of Matrine on migration of HPAC and Capan-1 cells were monitored by monolayer wound healing assay. Log-phase cells were seeded on six-well plates, and incubation with complete cell medium alone or contained with 50 μg/ml Matrine or with 0.5 μg/ml Docetaxel as indicated time. After wounded by a sterile 200 μl pipette tip, cells that were treated with normal cell medium migrated clearly. But the cells that were treated with Matrine or Docetaxel have limited migration (Fig. 1a, b and Additional file 1: Figure S1). In a three-dimensional cell migration assay with the transwell system, the invasion cell numbers of the group that treated with Matrine or Docetaxel for 10 h were less than the control group (Fig. 1c). This data indicated that the migration of HPAC cells was inhibited upon Matrine treatment via an unknown mechanism.

### Effects of Matrine on the expressions of MT1-MMP, MMP2, MMP9

**Fig. 2:**
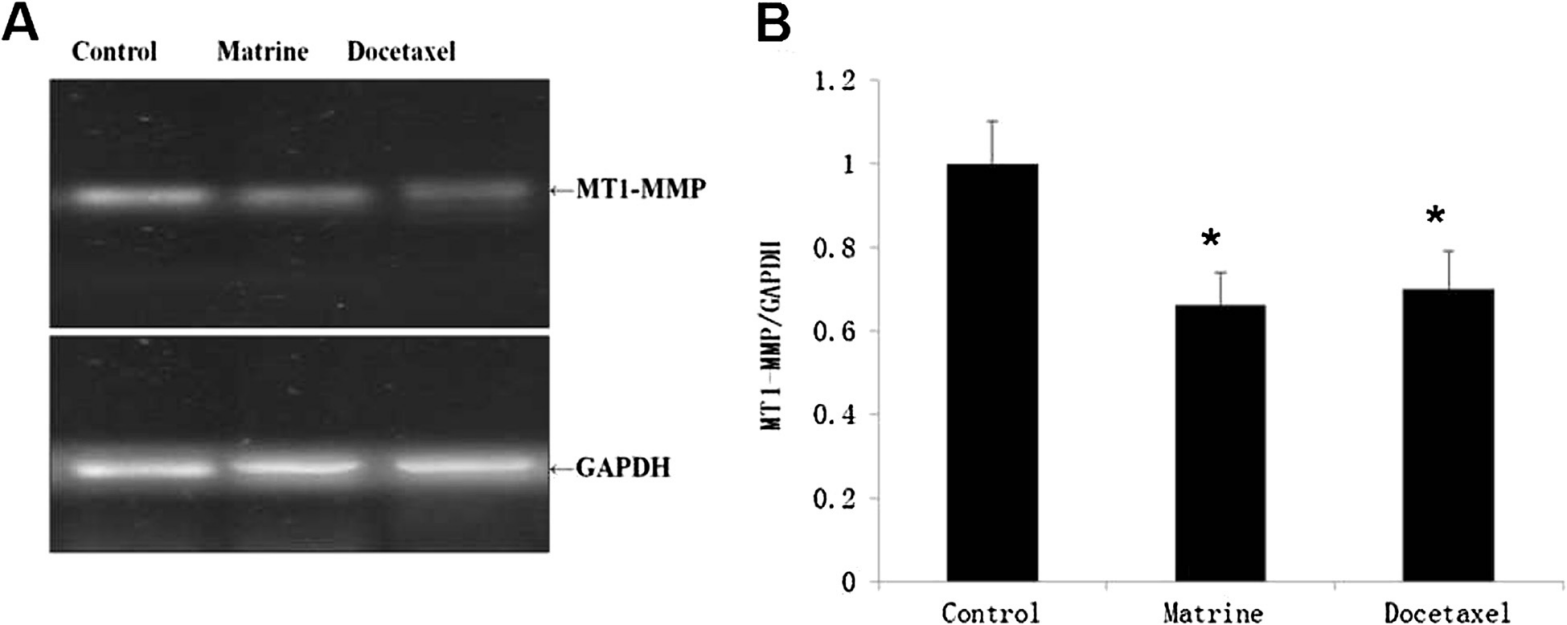
Matrine reduced the mRNA expression of MT1-MMP in HPAC cells. The mRNA expression of MT1-MMP in HPAC cell was analyzed by RT-PCR (**a**). The mRNA of GAPDH was used for internal control, that indicated the equal total mRNA. Data were expressed as mean ± S.E.M from three independent experiments. * (*P* < 0.05) indicates a significant difference compared with the control group (**b**)

**Fig. 3:**
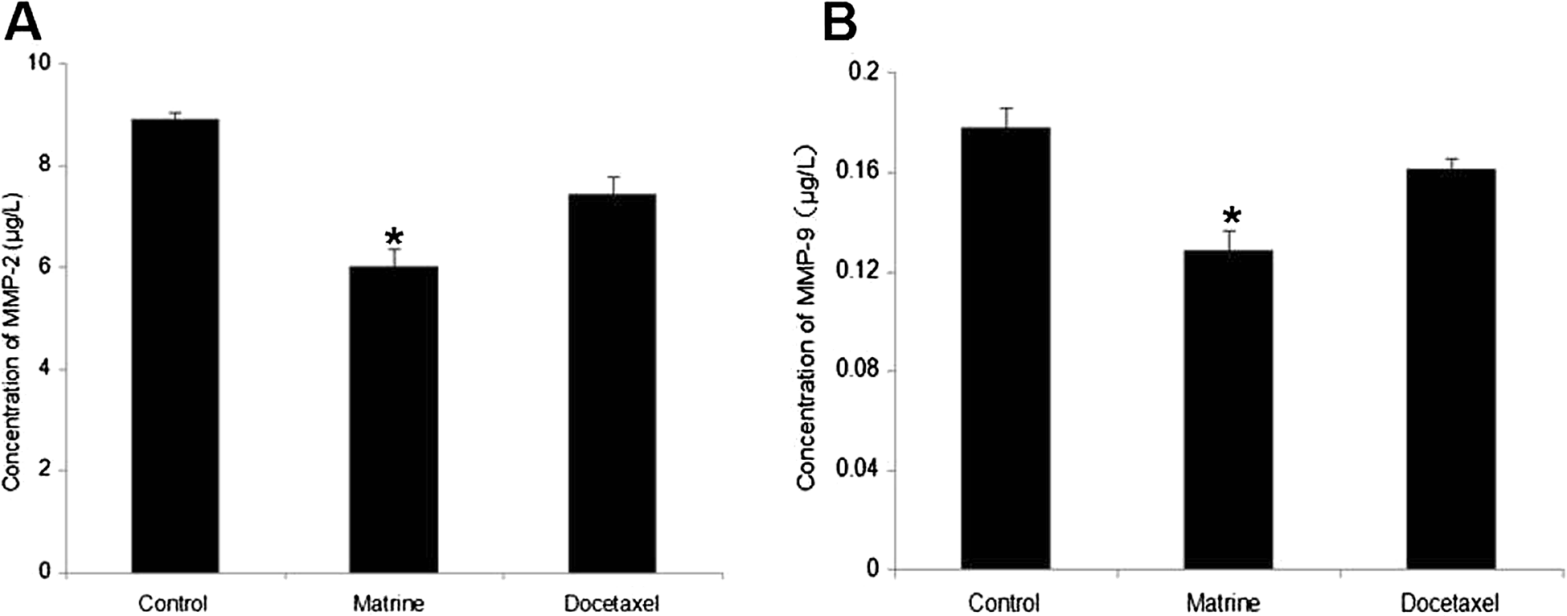
Effects of Matrine on the expressions of MMP2 and MMP9 in HPAC cells. HPAC cells were treated as described previously. The concentrations of MMP2 (**a**), MMP9 (**b**) in cell culture supernatant were analysed with ELISA assay. * (*p* < 0.05) indicates a significant difference compared with the control group

**Fig. 4:**
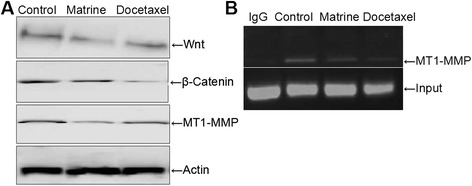
Effects of Matrine on the expressions of MT1-MMP, Wnt, β-catenin in HPAC cells. HPAC cells were treated as described previously. The expressions of Wnt, β-catenin and MT1-MMP were detected with western blot. Equal loading proteins were shown with β-actin immunoblot (**a**). The transcription activity of MT1-MMP in HPAC cells were detected by CHIP assay (**b**)

To explore the possible mechanism of the inhibition effect of Matrine on HPAC cells migration, we first detected the MT1-MMP expression level, which is the most important mediator of cell migration and invasion. RT-PCR was used to detect the expression of MT1-MMP in HPAC cells upon Matrine treatment. We found that MT1-MMP expression was decreased significantly upon Matrine treated cells (Fig. 2). Meanwhile, we detected the level of MT1-MMP protein upon Matrine treatment, as our expectation, MT1-MMP protein decreased evidently compared with the control group (Fig. 4a). We also detected the concentration of MMP2 and MMP9 in cell culture medium by ELISA kits, the results showed that the concentration of MMP2 and MMP9 decreased significantly in Matrine treatment (Fig. 3).

### Wnt signaling pathway may be involved in the MT1-MMP down-regulation by Matrine treatment

To further explore the exact mechanism of Matrine down-regulating MT1-MMP expression, we first investigated the effects of Matrine on the Wnt signaling pathway related properties. When HPAC cells were treated with Matrine for indicated time, the expression of Wnt and β-Catenin decreased significantly, paralleled with MT1-MMP expression level (Fig. 4a). These results suggested that the MT1-MMP expression was down regulated by Matrine maybe via Wnt signaling pathway.

### Effects of Matrine on MT1-MMP transcription activity via Wnt signaling pathway

CHIP assay was performed in order to determine the relationship between MT1-MMP transcription activity and Wnt signaling pathway. HPAC cells were treated with Matrine for 24 h, β-Catenin antibody was used for immunoprecipitated with target gene (Fig. 4b). The results have shown that MT1-MMP transcription activity decreased evidently compared with control cells, which demonstrated that Matrine regulated MT1-MMP expression through Wnt signaling.

## Discussion

Previous researches have indicated that Matrine has the potential function of inhibiting cancer cell migration, but the exact mechanism has not been unknown. Here, we have demonstrated that Matrine inhibited the migration and invasion of HPAC cells through down-regulation the Wnt-β-Catenin signaling pathway. In our present study, we first detected the cytotoxicity of Matrine on HPAC cell growth. We found that proliferation of HPAC cells were inhibited upon Matrine treatment, especially at too high a concentration or for too long a time (Additional file 2: Figure S2). The results are accordance with the earlier researches [2, 5]. In the next experiments, we have detected the invasion ability of HPAC or Capan-1 cells upon Matrine treatment through wound healing and transwell assays. We observed that the migration ability has been decreased significantly upon Matrine treatment for 24 h. Docetaxel was used for positive control to monitor the effect of cell migration inhibition. We found that there is no statistical significance between Matrine and Docetaxel treated groups.

Tumor invasion and metastasis were multistage and multi-factorial process, which were regulated by complicated mechanisms and multiple signaling pathways [18]. Many protein molecules have involved in regulating cancer cell adhesion, migration and invasion in tumor biology behaviors. Matrix metalloproteinases (MMPs) are a family of zinc-binding proteases that have been shown to contribute to cancer cell invasion through the ability to degrade ECM [19, 20]. MT1-MMP (also known as MMP-14) is the first identified and also the most common member of the MT-MMP subfamily involved in pericellular proteolysis associated with cell migration [21, 22].

In searching for the underlying mechanism of Matrine inhibited the migration and invasion of HPAC cells, we first detected the expression of MT1-MMP in HPAC cells upon Matrine treatment. Indeed, we found that the mRNA and protein expressions of MT1-MMP decreased evidently upon Matrine treatment.

The activities of most MMPs are very low in the normal tissues, however their expressions are regulated by various inflammatory cytokines, growth factors, hormones as well as by cell-cell interaction [23]. Moreover, the proteolytic activity of MMPs is strictly controlled at several levels, including transcriptional, posttranscriptional and post-translational, as well as via their endogenous inhibitors [24, 25]. The transcription of MT1-MMP was regulated by Wnt signaling pathway [26, 27], so we hypothesized that Matrine inhibited HPAC migration and invasion may be through down-regulation the Wnt signaling pathway. According to this assumption, we detected the expressions of Wnt and β-Catenin. Western blotting assay indicated that the expressions of Wnt and β-Catenin decreased markedly compared to the control group. However, the positive control, that the docetaxel treated group, their expressions decreased weakly. These results suggested that Matrine has a potential role in down-regulating the expressions of Wnt and β-Catenin. To investigate the transcription activity of MT1-MMP whether mediated by the Matrine treatment through Wnt signaling pathway, we detected the MT1-MMP transcription activity through CHIP assay. As our expected, upon Matrine treatment, a small amount of MT1-MMP was detected, decreased significantly compared with the control group.

In summary, our present data first demonstrated that Matrine play a potential role in inhibiting the migration and invasion of HPAC cells, which may be through down-regulation the expression of MT1-MMP via the canonical Wnt signaling pathway. It is interesting that Matrine down-regulation the expression of MT1-MMP, which is identified as an activator of proMMP-2 (pro-gelatinase A/72 kDa type IV collagenase) [28]. Since the degradation of the basement membrane by MMP-2 is likely a necessary step for cancer invasion [29, 30]. It is necessary to research whether Matrine mediated the activity of other MMPs, which is the next question we need to identify.

## Additional files

Additional file 1: Figure S1. Regulation of cell migration by Matrine in Capan-1 cells. Additional file 2: Figure S2. Regulation of cell proliferation by Matrine in HPAC or Capan-1 cells. Log-phase cells were treated with normal complete RPMI-1640 medium contained with various concentrations Marine for indicated time. Cell proliferation was detected with MTT assay. Data were expressed as mean ± S.E.M from three separate experiments. Statistical analysis was performed using the *t*-test. *(*P* < 0.05) indicates a significant difference compared with the control group.

## Abbreviations

CHIP: Chromatine immuno-precipitation
MMPs: Matrix metalloproteinases
