# Supplementary figures and images for: Effect of Matrine on HPAC cell migration by down-regulating the expression of MT1-MMP via Wnt signaling
Source: Cancer Cell Int. 2015 Jun 11;15:59. doi: 10.1186/s12935-015-0210-4 (PMC4480578; doi:10.1186/s12935-015-0210-4)

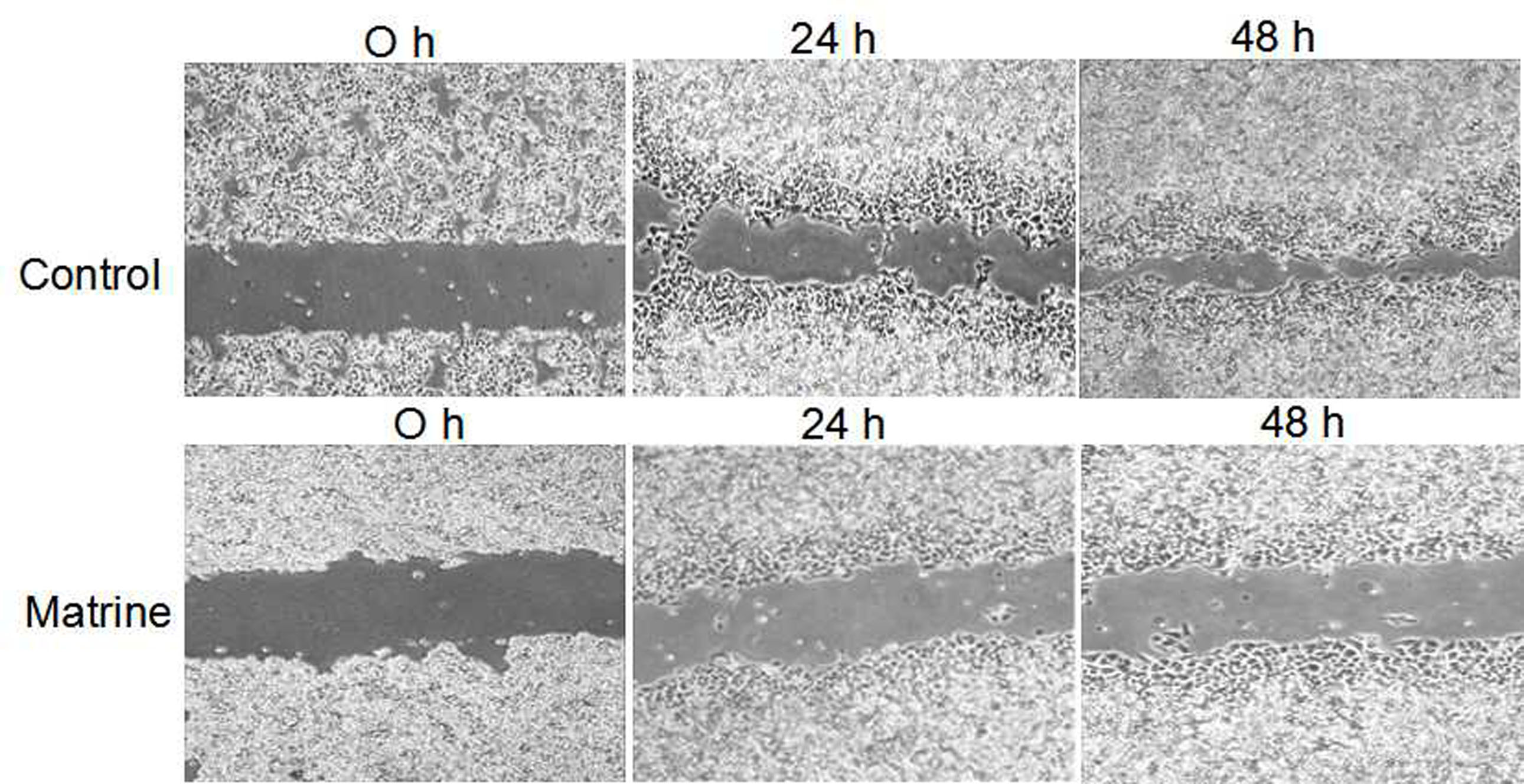

Supplement: Additional file 1: Figure S1. — Regulation of cell migration by Matrine in Capan-1 cells. [file 12935_2015_210_MOESM1_ESM.jpeg]

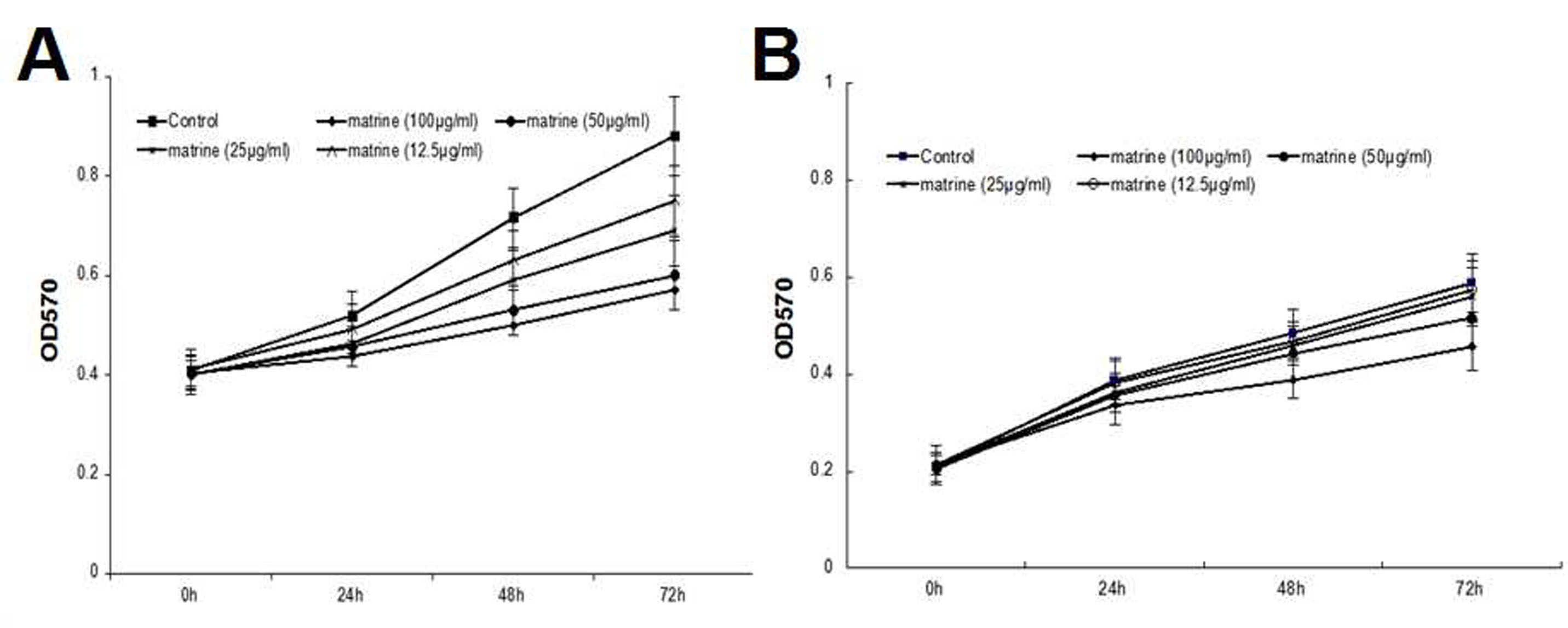

Supplement: Additional file 2: Figure S2. — Regulation of cell proliferation by Matrine in HPAC or Capan-1 cells. Log-phase cells were treated with normal complete RPMI-1640 medium contained with various concentrations Marine for indicated time. Cell proliferation was detected with MTT assay. Data were expressed as mean ± S.E.M from three separate experiments. Statistical analysis was performed using the t-test. *(P < 0.05) indicates a significant difference compared with the control group. [file 12935_2015_210_MOESM2_ESM.jpeg]
